# Intervention for bilingual speech sound disorders: A case study of an isiXhosa–English-speaking child

**DOI:** 10.4102/sajcd.v65i1.566

**Published:** 2018-03-19

**Authors:** Kate Rossouw, Michelle Pascoe

**Affiliations:** 1 Division of Communication Sciences and Disorders, Department of Health and Rehabilitation Sciences, Faculty of Health Sciences, University of Cape Town, South Africa

## Abstract

**Background:**

Bilingualism is common in South Africa, with many children acquiring isiXhosa as a home language and learning English from a young age in nursery or crèche. IsiXhosa is a local language, part of the Bantu language family, widely spoken in the country.

**Aims:**

To describe changes in a bilingual child’s speech following intervention based on a theoretically motivated and tailored intervention plan.

**Methods and procedures:**

This study describes a female isiXhosa–English bilingual child, named Gcobisa (pseudonym) (chronological age 4 years and 2 months) with a speech sound disorder. Gcobisa’s speech was assessed and her difficulties categorised according to Dodd’s (2005) diagnostic framework. From this, intervention was planned and the language of intervention was selected. Following intervention, Gcobisa’s speech was reassessed.

**Outcomes and results:**

Gcobisa’s speech was categorised as a consistent phonological delay as she presented with gliding of/l/in both English and isiXhosa, cluster reduction in English and several other age appropriate phonological processes. She was provided with 16 sessions of intervention using a minimal pairs approach, targeting the phonological process of gliding of/l/, which was not considered age appropriate for Gcobisa in isiXhosa when compared to the small set of normative data regarding monolingual isiXhosa development. As a result, the targets and stimuli were in isiXhosa while the main language of instruction was English. This reflects the language mismatch often faced by speech language therapists in South Africa. Gcobisa showed evidence of generalising the target phoneme to English words.

**Conclusions and implications:**

The data have theoretical implications regarding bilingual development of isiXhosa–English, as it highlights the ways bilingual development may differ from the monolingual development of this language pair. It adds to the small set of intervention studies investigating the changes in the speech of bilingual children following intervention. In addition, it contributes to the small amount of data gathered regarding typical bilingual acquisition of this language pair.

## Introduction

As throughout the world, multilingualism is typical in South Africa owing to a culturally and linguistically diverse population, as well as the mobility of the population and the country’s historical and political background. Many people speak two or more languages in their day-to-day life (Williams & Stroud, 2013). Although the country’s constitution states that all 11 official languages should be treated with equal esteem, and that children have the right to receive education in any of the official languages, many parents want their children to be educated in English rather than in their home language owing to the perceived higher status of English (Webb, Lafon & Pare, 2010). This article focuses on difficulties in the bilingual speech sound acquisition of isiXhosa and English.

IsiXhosa is one of the 11 official languages of South Africa. It is the second most widely spoken language in South Africa, with 16% of the population speaking it as a home language (Statistics South Africa, 2012). IsiXhosa is a Bantu language from the Nguni group along with isiZulu, SiSwati and isiNdebele. It is one of the main languages spoken in the Western Cape region of the country, along with Afrikaans and South African English.

Previous research into Bantu languages has considered various aspects of speech and language acquisition and disorders. For example, Demuth (2003) summarises research considering the acquisition of various Bantu languages, looking at the acquisition of noun classes, verbal morphology and syntactic structures and phonology. Suzman and Tshabalala (2000) considered the nature of language impairment in two isiZulu-speaking children, analysing their phonology, morphology, syntax and pragmatics. Demuth and Suzman (1997) also describe the language impairments displayed by an isiZulu-speaking child. Their findings suggested that the child’s language was qualitatively different from a typically developing isiZulu-speaking child’s language, and not just delayed.

Considering isiXhosa, the phonological development in isiXhosa-speaking children has been investigated (Gxilishe, 2004; Lewis & Roux, 1996; Maphalala, Pascoe & Smouse, 2014; Mowrer & Burger, 1991; Pascoe et al., 2016; Tuomi, Gxilishe & Matomela, 2001). Maphalala et al. (2014) conducted a cross-sectional study, investigating the speech production of 24 isiXhosa-speaking children between the ages of 3 and 6 years. An isiXhosa speech assessment, *Masincokoleni isiXhosa Speech Assessment* (Maphalala, Pascoe & Smouse, 2012) was developed to more accurately describe the speech skills of the children in the study. Results suggested that most consonants are acquired by the age of 3 years, with aspirated plosives, affricates, fricatives and clicks being amongst the later developing phonemes. Affricates were also identified as later developing by Tuomi et al. (2001) and Mowrer and Burger (1991). Mowrer and Burger (1991) also suggested that isiXhosa-speaking children acquire most consonants earlier than their English-speaking peers. This was similarly noted in two case studies conducted by Pascoe et al. (2016), who found that the two children in their study had acquired all of their vowels and many of their consonants by the ages of 2 years and 5 months, and 2 years and 8 months. Clicks, one of the most well-studied aspects of isiXhosa phonology, are acquired between the ages of 1 and 3 years (Gxilishe, 2004; Tuomi et al., 2001). Research has also described the typical simplifications used by young isiXhosa-speaking children as they acquire clicks (Lewis & Roux, 1996). IsiXhosa-speaking children make use of phonological processes for other consonants, most commonly gliding of liquids, stopping, depalatalisation, deaspiration and denasalisation (Maphalala et al., 2014).

Pascoe et al. (2015) considered the acquisition of English phonology by 3-year olds in Cape Town. Their sample included a small number (*n* = 25) of isiXhosa–English bilingual speakers and provides some data regarding the English phonology of isiXhosa–English bilingual 3-year olds. Findings suggested that the isiXhosa bilingual children in their sample had the most complete consonant inventories in English when compared with monolingual English speakers, bilingual English–Afrikaans speakers and trilingual English–Afrikaans–isiXhosa speakers in the study. However, the isiXhosa–English children also had the lowest mean percentage of correct vowels. The researchers questioned the validity of this score, suggesting it may have been caused by typical vowel substitutions used by isiXhosa–English bilingual speakers, given the complexity of the English vowel system in comparison to the five vowels of isiXhosa. The study also determined that the phonological processes of cluster reduction and stopping were more prevalent in the English speech of the isiXhosa–English children than in the other groups, as well as the processes of backing and devoicing (Pascoe et al., 2015), often considered non-developmental in monolingual English speech (Dodd, Holm, Hua & Crosbie, 2003). Backing and devoicing may be typical in the development of English by isiXhosa–English bilingual children. None of the isiXhosa–English bilingual children were identified as having a speech difficulty, although it is important to note that the sample of isiXhosa–English children was small (Pascoe et al., 2015).

It has been hypothesised that if a bilingual child presents with a speech sound disorder, the disorder will fall in the same category in both languages, suggesting that although the child has two phonological systems, a single underlying deficit affects both (Holm & Dodd, 1999b; Holm, Dodd, Stow & Pert, 1999). This seems to align with *interactional dual systems theory* (Paradis, 2001), which suggests a child’s two phonological systems interact in various ways as the child’s phonology develops, resulting in cross-linguistic transfer. This has been documented across a range of language pairs by authors, including Holm, Dodd, Stow and Pert (1997), Holm and Dodd (1999a, 1999b, 2001) and Ray (2002). Where a child presents with speech errors in only one language, this may be indicative of cross-linguistic transfer rather than a speech sound disorder (McLeod, Verdon & International Expert Panel on Multilingual Children’s Speech, 2017). For example, a child who speaks both English and French may not be able to produce the fricative [θ] in English and substitutes it with the plosive [t], as it is not present in French. If this use of the phonological process of stopping is only present in English, it is not necessarily caused by a speech sound disorder, but rather by cross-linguistic transfer from French to English. This highlights the importance of a speech language therapist (SLT) being able to identify whether a bilingual child presents with a speech sound disorder or difference (McLeod et al., 2017). A child acquiring two or more languages may present with differences in their speech that are not true errors but rather are caused by the typical acquisition of two languages or dialects (McLeod, 2012). This, too, must be differentiated from a child who presents with a true phonological disorder or delay (McLeod et al., 2017). In order to do this, knowledge of the typical development of bilingual children acquiring various language pairs is essential. For many language pairs, this information is lacking.

Although there have been a large number of studies considering bilingual intervention for language disorders, there are fewer that describe intervention for a bilingual child presenting with a speech sound disorder. A literature search identified seven articles that present an intervention study of bilingual or multilingual children with speech sound disorders that clearly describes the participants’ speech sound disorder, the intervention provided and the changes in the child’s speech following intervention (see Table 1). The studies presented vary in approach to intervention, as well as language of intervention, and investigated a variety of language pairs. The majority of the studies provided intervention only in English (Holm & Dodd, 1999a, 2001; Holm et al., 1997; Ray, 2002), while one provided intervention only in Arabic (Mamdouh, 2008) – in all of these cases, intervention was provided in the language that was dominant within the community. In contrast, two of the case studies provided intervention in both of the children’s languages, Spanish and English (Gildersleeve-Neumann & Goldstein, 2015) and Portuguese and English (Ramos & Mead, 2014). No intervention studies were found that include a language pair that is common amongst bilingual children in South Africa or a Bantu language.

**TABLE 1:** Summary of studies investigating the effect of intervention on the speech sound disorders of multilingual children.

Author/study	Languages	Participants	Language of therapy	Approach of therapy	Results
Holm et al. (1997)	Cantonese and English	Child aged 5 years and 2 months	English only, 15 weeks of intervention	7 weeks of articulation intervention (20 min, twice a week), 8 weeks of phonological therapy (45 min once a week, using phonological contrasts)	Cross-linguistic generalisation occurred for articulation targets but not phonological targets
Holm and Dodd (1999b)	Punjabi and English	Child aged 4 years and 6 months, inconsistent speech sound disorder in both languages	English only 16 sessions of 30 min duration over 8 weeks	Core vocabulary, including parent training	Increased consistency of productions of treated and untreated words in English. Smaller increase in consistency in Punjabi
Holm and Dodd (2001)	1.Cantonese and English	Child aged 5 years and 2 months	English only, 15 weeks	7 weeks of articulation intervention (20 min, twice a week), 8 weeks of phonological therapy (45 min, once a week)	Cross-linguistic generalisation occurred for articulation target, but not phonological targets
	2.Punjabi and English	Child aged 4 years and 8 months, inconsistent disorder	English only, 16 sessions of 30 min duration over 8 weeks	Core vocabulary	Cross-linguistic generalisation occurred
Ray (2002)	Hindi, Gujarati and English	5-year-old child, similar developmental but not age appropriate phonological process in all three languages, with a small amount of inconsistency (10% – 30%)	English only, 40 sessions of 45–60 min over 20 weeks	Cognitive linguistic approach: minimal contrast therapy, focusing on both perception and production of contrasts. Included parent training	Increased PCC; increased intelligibility; decreased use of phonological processes. Generalisation to all languages. Residual errors in conversational speech
Mamdouh (2008)	Arabic and English	5-year-old child, with ‘delayed language affecting phonology’ (Mamdouh, 2008, p. 38)	Arabic only, 43 sessions of 30 min duration, twice a week over 7 months	Intervention was structured in four steps, targeting different phonemes. Intervention included description of the characteristics of the phonemes; sensori-perceptual training; production of the sound in isolation, syllables, words, phrases, sentences and spontaneous speech	PCC improved in both Arabic and English after most steps; however, his English PCC did not improve after the step that focused on phonemes specific to Arabic (/ħ/and/x/). The use of/v/in English also improved, even though not present in Arabic or targeted in intervention
Ramos and Mead (2014)	Portuguese and English	Sequential bilingual child aged 6 years and 5 months with a severe speech sound disorder	Three intervention phases, each lasting 2 months: English and Portuguese by two therapists, focusing on different targets in each language. 1 h sessions, twice a week, by each therapist, resulting in 4 h of intervention each week English and Portuguese provided by one therapist. 1 h a week in English, 1 h a week in Portuguese English only. 1 h, twice a week	Auditory discrimination training; production in isolation, syllables, words, phrases; minimal pair activities included in drill play	Although progress was noted throughout, the most progress was noted in the phase providing bilingual Portuguese–English intervention. Bidirectional transfer occurred when targeting phonemes with similar rules in both languages
Gildersleeve-Neumann and Goldstein (2015)	Spanish and English	Children aged 5 years and 8 months and 5 years and 6 months, respectively; one with a moderate SSD, one diagnosed with childhood apraxia of speech	Spanish and English: Intervention provided 2–3 times a week, in Spanish at least 2 out of every 3 days. A total of 19 and 25 sessions, respectively, were reported on	Combined the following features: (1) meta- and perceptual awareness of session goals and how they linked to both languages; (2) developmentally appropriate activities to facilitate drill play; (3) articulatory and phonological components and cueing; (4) practicing targets in functional utterances	Increases in accuracy of targets and overall accuracy in both languages

PCC, percentage consonants correct.

The procedures and approaches employed in the studies varied greatly, resulting in difficulty drawing conclusions. However, it is interesting to note the findings of Gildersleeve-Neumann and Goldstein (2015) and Ramos and Mead (2014) who investigated the effect of providing intervention in both languages. The use of both the child’s languages in intervention is more in line with the recommendations provided by various individuals and organisations (e.g. Gildersleeve-Neumann & Goldstein, 2012; Goldstein & Fabiano, 2007; International Expert Panel on Multilingual Children’s Speech, 2012). Results indicated an increase in overall accuracy in both languages (Gildersleeve-Neumann & Goldstein, 2015; Ramos & Mead, 2014). The results recorded by Ramos and Mead (2014) are worth mentioning, as they compared the results of a period of intervention in one language (English) to those following a period of intervention in two languages (English and Portuguese). They concluded that for their participant, even though some improvement of specific phonological processes was noted in Portuguese when the processes were targeted in English, there was a much greater improvement when she received bilingual intervention (1 h of English and 1 h of Portuguese per week). In addition, although providing English intervention improved her English speech production, bilingual intervention had a greater effect on improving her English production. However, they did note that some phonological processes (e.g. fronting of palatal fricatives) had to be specifically targeted in Portuguese, her weaker language, for the child to show indications of eliminating those specific processes from her Portuguese speech (Ramos & Mead, 2014).

This study aimed to describe changes occurring in an isiXhosa–English bilingual child’s speech following intervention targeted at her speech sound difficulties. We intended to add to the limited research considering intervention for speech sound disorders with bilingual children, as well as considering the challenges of assessing the speech of a bilingual child when little is known about the typical bilingual speech development of the language pair spoken by the child.

## Method

The objective of this study was to analyse and describe the isiXhosa and English speech of a bilingual isiXhosa–English-speaking child before and after providing intervention based on a tailor-made, theoretically motivated intervention plan. It followed an exploratory descriptive design in order to detail the changes that occurred in the child’s speech. A single subject pre-test and post-test design was used: this allowed the participant to be described as an individual case and act as her own control (Vance & Clegg, 2012), and included assessing the child’s speech before and after the intervention.

### Participant

IsiXhosa–English bilingual children with possible speech sound disorders were referred to the researcher by teachers at local crèches. After consent was obtained from their parents, these children were assessed, and the participant was randomly selected from the smaller pool of children who met the criteria: between the ages of 3 and 6 years, bilingual with isiXhosa as their home language and English as an additional language, presented with a speech sound difficulty as their primary difficulty and had not received previous intervention.

Gcobisa^1^, an isiXhosa–English bilingual girl was 4 years and 2 months at the start of the study. She was referred to the researcher by her teacher owing to concerns regarding her speech development. Gcobisa’s mother gave consent for Gcobisa to take part in the study. Gcobisa’s mother reported that her child had been exposed to both isiXhosa and English from a young age, both at home and at school, as well as some Southern Sotho, another local language, at home. She reported her motor milestones were in the average range, while her speech and language milestones appeared slightly delayed.

### Assessment

English speech was assessed using the *Diagnostic Evaluation of Articulation and Phonology* (*DEAP*) (Dodd, Hua, Crosbie, Holm & Ozanne, 2002). This is an assessment tool developed in the United Kingdom that assesses a child’s articulation, phonology, inconsistency and oral motor skills in English and allows for clear descriptions and categorisation of speech sound disorders. IsiXhosa speech was assessed using the *Masincokoleni isiXhosa Speech Assessment* (Maphalala et al., 2012), a single-word naming test developed in South Africa to evaluate a child’s phonology in isiXhosa. Receptive language in both English and isiXhosa was assessed using the *Peabody Picture Vocabulary Test* (Fourth Edition) (*PPVT-4*) (Dunn & Dunn, 2007), using an isiXhosa translation of the *PPVT-4* developed by Dawes, Biersteker and Hendricks (2012). These tests assess a child’s understanding of vocabulary (single words) and give an indication of their understanding of each language. As the isiXhosa version was a translation, the results were analysed descriptively. All assessment sessions were audio recorded. A portion of the audio recorded data was transcribed both by the researcher, and a linguist familiar with both isiXhosa and English. Thus, intra-rater and inter-rater reliability were established to ensure data analysis was based on accurate data.

The results of these assessments were analysed to place the participant’s speech sound disorder into a functional category as described by Dodd (2005). From this, an intervention plan was developed and implemented (see results for further details on how this plan was developed). At each session, a fidelity checklist was completed to ensure the intervention followed the intervention plan in terms of approach of intervention and language of intervention. Following intervention, the participant’s English and isiXhosa speech were reassessed, using the same assessment tools.

## Ethical consideration

The study received ethical clearance from the University of Cape Town Faculty of Health Science Human Research Ethics Committee, HREC REF: 448/2015.

## Results

### English speech assessment

Gcobisa’s pre-intervention assessment results are summarised in Table 2.

**TABLE 2:** Gcobisa’s initial assessment results.

Area assessed	English	isiXhosa
Speech inventory (missing sounds)	[θ, ð, ɹ]	[r, kx, c^h^, ɮ, tɬ, ts^h^ and tʃ^h^]
PCC^a^	75%	77%
PVC^a^	98%	96%
PPC^a^	83%	88%
Phonological processes	Gliding, cluster reduction	Gliding, backing of palatal plosive
Inconsistency (percentage of words produced differently on repeated production)	28%	14%
Oro-motor skills	Age appropriate	-
Language (receptive)	RS 25	RS 22

a , PCC was calculated by dividing the number of correctly produced consonants by the total number of consonants produced. PVC was calculated by dividing the number of correctly produced vowels by the total number of vowels produced. PPC was calculated by dividing the number of correctly produced phonemes by the total number of phonemes produced.

PCC, percentage consonants correct; PPC, percentage phonemes correct; PVC, percentage vowels correct; RS, raw score.

Gcobisa’s phonetic inventory was age appropriate (see Appendix 1, Table 1-A1). Although the fricatives [θ] and [ð] and the phoneme [ɹ] were missing from her inventory, this is age appropriate in comparison to monolingual norms (Dodd et al., 2002). All other English sounds were present in her phonetic inventory. In addition, Gcobisa showed evidence of being able to produce all vowels and diphthongs appropriately.

Gcobisa used many developmental phonological processes and few non-developmental processes (see Table 2), but most of them were isolated occurrences (not used consistently on every production of the phoneme), suggesting that she is in the process of eliminating many of them. This made her speech seem unintelligible and occasionally inconsistent, and she had a percentage consonants correct (PCC) of 75% as a result of her errors. However, the results of the inconsistency assessment suggested her speech did not meet the criteria for an inconsistent speech disorder. Gcobisa has two consistent phonological processes: gliding and cluster reduction. The process of gliding was still age appropriate as it should be eliminated by the age of 5 years and 11 months according to monolingual English norms (Dodd et al., 2002). The process of cluster reduction of two part clusters was of more concern, as a monolingual child acquiring English should be able to produce clusters containing two consonants by the age of 3 years and 11 months (Dodd et al., 2002). From this information, it was determined that Gcobisa presented with a mild phonological delay in English.

### IsiXhosa assessment

Gcobisa’s isiXhosa phonetic inventory was considered age appropriate, although she was not yet producing the phonemes [r] and [kx], which she should be in the process of acquiring (see Appendix 1, Table 2-A1). She was able to produce all isiXhosa vowels accurately with very few errors. Her PCC and percentage phonemes correct (PPC), however, were low in comparison to the scores described by Maphalala et al. (2014), although they were similar to her English PCC and PPC scores.

As with her English speech, she made use of some phonological processes consistently, as well as some on isolated occurrences. This made her speech seem inconsistent, but comparing her productions of the same word, her consistency was judged to be appropriate for her age.

Two consistent phonological processes were gliding and backing of the palatal plosive. According to the information gathered in the study by Maphalala et al. (2014), the process of gliding was found to have been eliminated in the speech of the children by the age of 3 years and 6 months. Considering this, Gcobisa’s use of the phonological process of gliding could be considered delayed. It was determined Gcobisa presented with a mild phonological delay in isiXhosa.

Her receptive language scores suggested her receptive language skills were not yet at an age-appropriate level. However, her understanding of language was judged to be adequate for participation in therapy activities.

### Intervention plan

Gcobisa had a mild phonological delay in both English and isiXhosa. The minimal pairs approach was selected as an appropriate approach for this type of speech difficulty (Baker, 2010).

Intervention aims included:

Gcobisa will be able to produce [l] accurately:

- in initial position, for example, in verbs that, when produced as a command, start with consonants such as *luma* (bite)
- and medial position, for example, after the prefix of nouns, such as *ilanga* (sun)

in words and in phrases in isiXhosa.

The language of instruction throughout all intervention sessions was English. This was because of a variety of reasons: Gcobisa showed evidence of understanding English to the same degree or more than isiXhosa; in addition, as a language mismatch is common between SLTs and clients in South Africa, this reflected the realities of most SLTs currently providing intervention in South Africa. However, the language of the intervention stimuli was considered further. Although the process of gliding was present in both English and isiXhosa, gliding was still considered to be an age-appropriate process for Gcobisa in English. As such, all targets chosen were in isiXhosa, as the process of gliding is not age appropriate for Gcobisa in isiXhosa.

### Intervention

Gcobisa attended 16 sessions of intervention over 8 weeks, attending sessions lasting 30 min, twice a week. Intervention took place in a quiet room at her crèche. She received approximately 8 h of intervention. Each session was guided by a fidelity checklist to ensure sessions adhered to the intervention developed for Gcobisa’s speech sound disorder. Gcobisa was initially shy but soon began participating more in sessions.

The following steps, based on those outlined by Baker (2010), were followed:

- familiarise Gcobisa with the minimal pair pictures and words
- train Gcobisa to perceive the difference between the minimal pairs
- imitation of target words
- production of minimal pairs, highlighting communication breakdown where appropriate
- generalisation activities.

### Post-intervention English speech assessment

Following intervention, Gcobisa’s speech was reassessed. Results are summarised in Table 3.

**TABLE 3:** Summary of post-intervention English speech assessment results.

Area assessed	Initial assessment (4 years and 2 months)	Reassessment (4 years and 6 months)
Inventory	Incomplete but age-appropriate (missing [θ, ð, ɹ])	Complete
PCC in phonology assessment	75%	79%
Number of times error patterns used in phonology assessment (5 or more instances):		
Gliding	13	5
Cluster reduction	10	9
Total number of errors in phonology assessment (including isolated processes)	34	24
Inconsistency in DEAP screener	50%	20%
Inconsistency assessment	24%	8%

DEAP, Diagnostic Evaluation of Articulation and Phonology.

Following intervention, Gcobisa’s phonetic inventory was age-appropriate with the fricatives [θ] and [ð] present. Gcobisa still used many developmental phonological processes and a few non-developmental processes, but most of them were isolated occurrences, suggesting that she is in the process of eliminating many of these phonological processes. In the phonology subtest, Gcobisa’s PCC improved from 75% to 79%. Her use of gliding reduced from 13 to five instances and her overall inaccuracies from 34 to 24 instances. Gcobisa still made use of the two consistent phonological processes: gliding and cluster reduction. However, gliding of [l] only accounted for two of the five instances of gliding, the remaining three being instances of gliding [ɹ]. The process of gliding is still age-appropriate as it should be eliminated by the age of 5 years and 11 months (Dodd et al., 2002). She made use of the phonological process of cluster reduction at a similar level as the initial assessment. Her use of cluster reduction continues to be of concern as, considering monolingual English norms, she should have eliminated this phonological process by the age of 3 years and 11 months (Dodd et al., 2002). Her speech was more consistent, with an inconsistency of only 8% in comparison to 24% in the initial assessment. As Gcobisa still made use of the immature process of cluster reduction, she still presented with a mild phonological delay.

### Post-intervention isiXhosa speech results

Gcobisa’s isiXhosa reassessment results are summarised in Table 4. Gcobisa’s inventory increased, with the only immature omission being [r] (Maphalala et al., 2014). Her PPC, percentage vowels correct (PVC) and PPC all increased and were considered age appropriate in comparison to the small sample of data collected for *Masincokoleni* (Maphalala et al., 2014). Although she did still make use of some phonological processes, the majority of these were isolated occurrences, and on many occasions she was able to produce the accurate production of the word on the second attempt. Gcobisa’s speech was more consistent, with only two instances of inconsistent productions of words, and both of these contrasting an inaccurate production with an accurate production of the word.

**TABLE 4:** Summary of post-intervention isiXhosa speech assessment results.

Area assessed	Initial assessment (4 years and 2 months)	Reassessment (4 years and 6 months)
Inventory	Incomplete and not age-appropriate [phonemes that Gcobisa should have acquired: (ɮ, |g, r)]	Incomplete and not age-appropriate [phoneme that Gcobisa should have acquired: (r)].
PCC in phonology assessment	77%	94%
Number of times error patterns used in assessment:		
Gliding	9	1
Backing of palatal plosives	2	2

PCC, percentage consonants correct.

Gcobisa only made use of gliding the trill [r]. Although this process should be eliminated by her age, the phoneme [r] is not common in isiXhosa. On two occasions, she made use of the process of backing of the palatal plosives. Although this is usually considered a non-developmental process, Pascoe et al. (2015) suggest that this may be a typical process for isiXhosa–English bilingual children in Cape Town. Gcobisa’s use of gliding of [r] suggested that she still had a mild phonological delay.

## Discussion

This study considered the changes in a bilingual child’s speech in both languages when provided with intervention targeting her speech sound disorder. The intervention approach of minimal pairs was chosen to target Gcobisa’s speech sound disorder (Baker, 2010), resulting in a change in Gcobisa’s phonological system, as she was able to accurately produce the phoneme [l] in both trained and untrained words and phrases in isiXhosa and in words in English.

Kohnert (2010) highlights three issues that clinicians need to take into account when considering bilingual children regardless of whether they have acquired their languages simultaneously or sequentially: (1) children may present with an uneven distribution of skills in their two languages, so their language skills may be distributed across both languages rather than duplicated from their stronger language to their weaker language; (2) some form of cross-linguistic interaction will take place; and (3) owing to the complex interaction between personal factors evident in all children (e.g. socioeconomic situation and general exposure to language at home) as well as factors specific to bilingual children (e.g. exposure to each language, age of acquisition of each language and opportunity to use each language), bilingual children present as a very heterogeneous group, making it difficult to compare a bilingual child’s development to norms, even when those norms are based on a similar group of bilingual children. This was evident in the case under discussion, and the lack of information regarding typical development in the isiXhosa–English population resulted in challenges in interpreting the data in the current study. Interpretation was based on monolingual norms, but this is problematic, as bilingual speech development is known to differ from the monolingual development of the languages (Holm & Dodd, 2006). This results in bilingual children often being under-referred or over-referred for intervention (Hambly, Wren, McLeod & Roulstone, 2013), as demonstrated in the current study. Although Gcobisa’s teachers were eager to refer her to the researcher and reported concerns regarding intelligibility, detailed analyses of phonological skills were difficult, as little is known about isiXhosa–English bilingual acquisition. In addition, her mother did not share the teacher’s concerns. Results were compared to monolingual norms, and the effect on Gcobisa’s participation in the classroom guided the decision to provide intervention. However, if one considers that Gcobisa is acquiring both English and isiXhosa, and this acquisition is different to monolingual development of each language, her speech development may be typical for a bilingual child, particularly considering the heterogeneity of the bilingual population.

Preliminary research into the typical development of South African English has included a small sample of isiXhosa–English bilingual children (*n* = 25) and provided some information regarding their English speech development (Pascoe et al., 2015). One of the findings in that study suggested that backing, a phonological process considered non-developmental in English, was prevalent in the English speech of isiXhosa–English bilingual participants. Gcobisa showed evidence of backing in isiXhosa. This adds to the data collected by Pascoe et al. (2015), adding to the evidence that backing may be a typical process in bilingual isiXhosa–English children. However, unlike Pascoe et al. (2015), this study also considered isiXhosa speech. Backing was evident in Gcobisa’s isiXhosa speech but was not evident in the monolingual isiXhosa sample under consideration by Maphalala et al. (2014). This process may be considered typical when evident in one or both languages of isiXhosa–English bilingual children, highlighting a difference between monolingual and bilingual speech acquisition of the two languages. However, a larger sample of isiXhosa–English children is required to determine whether this is typical of the larger population, as previous research has focused on fairly small samples.

Another process identified as being more prevalent in isiXhosa–English bilinguals than in monolinguals is cluster reduction (Pascoe et al., 2015). This may be the result of the effect of one language on the other: isiXhosa makes use of very few clusters (Maphalala et al., 2014). It is therefore not surprising that children who speak isiXhosa as a home language find the production of clusters difficult, often reducing them. This was noted in the English speech of Gcobisa. Cluster reduction is expected to be eliminated from the speech of monolingual English children by the age of 3 years and 11 months (Dodd et al., 2003) and yet this was the most prevalent process in the speech of Gcobisa (4 years and 6 months at the end of the study). This suggests that it may be typical for isiXhosa–English children to continue to use cluster reduction beyond the age of 4 years. However, again, this would need to be explored further with a larger sample of isiXhosa–English bilingual children.

South Africa’s multilingual environment creates challenges for SLTs. The current demographic of qualified SLTs is poorly representative of the South African population, as the majority of SLTs speak English and/or Afrikaans (Pascoe & Norman, 2011). Although this is gradually changing to reflect a more diverse demographic, there is often a language mismatch between SLT and client (Pascoe & Norman, 2011). Globally, this is also a common problem as discussed in a tutorial article by McLeod et al. (2017) entitled ‘Speech assessment for multilingual children who do not speak the same language(s) as the speech language pathologist’. Around the world, SLTs face further challenges in providing appropriate intervention for children who are multilingual, as there is limited research on the appropriate approach for intervention for this population (Goldstein & Fabiano, 2007).

In the recruitment process, a number of bilingual children were referred to the researcher and assessed. However, after analysing their speech using monolingual norms, very few of these children presented speech sound disorders. In South Africa, a preliminary study investigating the speech of 150 3-year-old children acquiring English identified that 6.66% of the children presented with speech sound disorders (Pascoe et al., 2015). Of the 6.66%, none were isiXhosa–English bilingual children. Further research into the speech development of bilingual children, particularly those acquiring isiXhosa and English, would be useful in order to aid SLTs in being able to differentiate between a speech disorder and speech differences.

Many previous studies considering intervention for bilingual children provided intervention in only one of the multilingual child’s languages (Holm & Dodd, 1999a, 2001; Holm et al., 1997; Mamdouh, 2008; Ray, 2002). Ramos and Mead (2014), however, provided intervention in both languages of a bilingual child. From their results, they concluded that intervention in both languages was more effective than intervention provided in only one of the child’s languages, even if generalisation takes place (Ramos & Mead, 2014). Considering the case of Gcobisa, although the targets chosen were in isiXhosa, the main language used within the sessions was English, with some simple isiXhosa instructions [e.g. *hlala pantsi* (sit down); *mamela* (listen); *ewe* (yes)]. This was a situation not well-documented in the literature, where the targets were in isiXhosa while the instructions and other interactions with the researcher took place in English. This may have had an impact on the generalisation from the treated isiXhosa to the untreated English.

Holm et al. (1997), Holm and Dodd (2001) and Mamdouh (2008) reported situations where generalisation did not occur from the treated to the untreated language. In these cases, the targeted error or phoneme appeared to be specific to the targeted language or uncommon in the untreated language. This would suggest that if a targeted error pattern or phoneme is common to both languages, intervention in one language may result in generalisation of results to the untreated language, as evidenced in Gcobisa’s results. Gcobisa made use of gliding of [l], producing the liquid as [j] in both English and isiXhosa. Intervention that used targets from only one language (isiXhosa) resulted in generalisation of results to the untreated language (English), as Gcobisa showed evidence of eliminating this phonological process from both her isiXhosa and her English speech.

## Clinical implications

This case study considered the changes in the speech of a bilingual child following intervention. Intervention sessions were conducted predominantly in English, while targets and stimuli chosen were in isiXhosa. These unique conditions appear to have resulted in change to the phonology of both languages. The language mismatch between the researcher and the child mirrors the situation often experienced by South African SLTs, who are not always able to speak all of the languages spoken by their clients. Although providing intervention in both languages is considered ideal (Gildersleeve-Neumann & Goldstein, 2012; Goldstein & Fabiano, 2007; International Expert Panel on Multilingual Children’s Speech, 2012), in situations where this is not possible and translators are not readily available, the use of targets in one language and intervention in the other language may result in change to both languages, although further research is necessary with a larger sample of children to generalise this to the wider population. Finally, this article also added to the small body of research considering isiXhosa–English typical speech acquisition. The child in the single case study presented with backing and cluster reduction that persisted beyond the typical age of elimination, both processes also noted by Pascoe et al. (2015) in the English speech of the isiXhosa–English bilingual children included in their study. This adds to the evidence that these processes may be typical in the acquisition of isiXhosa–English speech.

## Footnotes

1 A pseudonym was used to ensure anonymity.

## Appendix 1:

### Gcobisa’s consonant inventories in English and isiXhosa

**TABLE 1-A1:** Gcobisa’s English consonant inventory.

Manner	Bilabial	Place					
		Dental-Labial	Interdental	Alveolar	Palatal	Velar	Glottal
Plosives	p, b	-	-	t, d	-	k, g	-
Nasal	m	-	-	n	-	ŋ	-
Fricative	-	f, v	-	s, z	ʃ, ʒ	-	h
Glide	w	-	-	-	j	-	-
Affricate	-	-	-	-	tʃ, ʤ	-	-
Liquid	-	-	-	I	-	-	-

**TABLE 2-A1:** Gcobisa’s isiXhosa consonant inventory.

Manner	Place						
	Bilabial	Dental-Labial	Alveolar	Prepalatal	Palatal	Velar	Glottal
**Plosives:**							
Ejective	p’	-	t’	-	c’	k’	-
Aspirated	p^h^	-	t^h^	-	-	k^h^	-
Voiced	b	-	d	-	ï	g	-
**Implosive:**							
Voiced	ɓ	-	-	-	-	-	-
**Nasal:**							
Voiced	m	-	n	ɲ	-	ɳ	-
**Fricative:**							
Unvoiced	-	f	s	-	ʃ	x	h
Voiced	-	v	z	-	-	ɣ	-
Aspirated lateral	-	-	ɬ	-	-	-	-
Voiced lateral	-	-	-	-	-	-	-
**Approximant:**							
Lateral	-	-	l	-	-	-	-
Voiced glides	w	-	j	-	-	-	-
**Trill:**							
Voiced	-	-	-	-	-	-	-
**Affricate:**							
Ejective	-	-	ts’	tʃ’	-	-	-
Aspirated	-	-	-	-	-	-	-
Voiced	-	-	dz	d_Ʒ_	-	-	-
Voiceless	-	-	-	-	-	-	-

**TABLE 3-A1:** Gcobisa’s inventory of clicks.

Place	Click only	Aspirated	Regular nasal	Breathy nasal	Voiced
Dental	ǀ	ǀʰ	ŋǀ	ŋǀ	-
Alveolar	!	!ʰ	ŋǃ	ŋǃ	ǃɡ̊
Lateral	ǀǀ	ǀǀʰ	ŋǀǀ	ŋǀǀ	ǀǀɡ̊
